# Supplementary material for: Structural basis for PRC2 decoding of active histone methylation marks H3K36me2/3
Source: eLife. 2020 Nov 19;9:e61964. doi: 10.7554/eLife.61964 (PMC7725500; doi:10.7554/eLife.61964)
Supplement: Supplementary file 1. [file elife-61964-supp1.docx]

**Supplementary file 1**

| **Cryo electron microscopy data collection (applicable to Overall PHF1-PRC2:diNuc and EZH2_sub_-Nuc_sub_ maps)** | | |
| --- | --- | --- |
| Microscope | FEI Titan Krios GII | |
| Voltage (kV) | 300 | |
| Camera | Gatan K2-Summit | |
| Energy Filter | Gatan Quantum-LS (GIF) | |
| Pixel size (Å/pix) (calibrated) | 1.75 | |
| Nominal magnification (x) | 81000 | |
| Preset target global defocus range (μm) | 0.5 - 3.5 | |
| Total electron exposure (fluence, e^-^/Å^2)^ | 52,96 | |
| Exposure rate (flux) (e^-^/ Å^2^/s) | 3,47 | |
| Nr. of frames collected per micrograph | 60 | |
| Energy filter slit width (eV) | 20 | |
| Automation Software | SerialEM | |
| **3D reconstruction (applicable to Overall PHF1-PRC2:diNuc and EZH2_sub_-Nuc_sub_ maps)** | | |
| Number of movies | 3466 | |
| Initially selected particle candidates | 1,028,229 | |
| Final number of particles | 45,849 | |
|  | **Overall PHF1-PRC2:diNuc**  **(EMD-11912)** | **EZH2_sub_-Nuc_sub_**  **(EMD-11910)** |
| Resolution _FSC independent halfmaps (0.143) masked_ (Å)**^a^** | 5.24 | 4.36 |
| Local resolution range (Å) | 4.01 - 24.97 | 4.01 – 15.00 |
| Sharpening B-factor (Å^2^) | -90.7 | -76.5 |
| **Refinement EZH2_sub_-Nuc_sub_** |  | **EZH2_sub_-Nuc_sub_**  **(PDB 7AT8)** |
| No. atoms |  | 29151 |
| Residues |  | Protein: 1186 Nucleotide: 312 |
| Ligands |  | ZN: 7 |
| CC_mask,_ CC_box_, CC_peaks_, CC_volume_**^b^** |  | 0.75, 0.83, 0.66, 0.74 |
| Mean CC for ligands |  | 0.71 |
| Resolution_FSC masked map vs. model (0/0.143/0.5)_ (Å)**^b^** |  | 4.3/4.3/4.6 |
| R.m.s. deviations |  |  |
| Bond lengths (Å) |  | 0.004 |
| Bond angles (°) |  | 0.828 |
| Ramachandran favored (%) |  | 97.49 |
| Ramachandran gen. allowed (%) |  | 2.25 |
| Ramachandran disallowed (%) |  | 0.26 |
| MolProbity score |  | 1.52 |
| Clash score |  | 7.65 |
| ADP (B factors) |  |  |
| Iso/Aniso (#) |  | 15955/0 |
| Min/max/mean |  |  |
| Protein |  | 30.00/254.73/119.01 |
| Nucleotide |  | 95.54/222.73/130.75 |
| Ligand |  | 150.31/257.19/166.85 |
| Rotamer outliers (%) |  | 0.40 |
| Cβ outliers (%) |  | 0.00 |
| CaBLAM outliers (%) |  | 1.41 |
|  |  |  |
|  |  |  |

**^a^**according to the Fourier Shell Correlation (FSC) cut-off criterion of 0.143 defined in (Rosenthal and Henderson, 2003)

**^b^**according to the map-vs.-model Correlation Coefficient definitions in (Afonine et al., 2018a)
