# Supplementary material for: Structural basis for PRC2 decoding of active histone methylation marks H3K36me2/3
Source: eLife. 2020 Nov 19;9:e61964. doi: 10.7554/eLife.61964 (PMC7725500; doi:10.7554/eLife.61964)
Supplement: Transparent reporting form [file elife-61964-transrepform.docx]

***eLife’s* transparent reporting form**

We encourage authors to provide detailed information *within their submission* to facilitate the interpretation and replication of experiments. Authors can upload supporting documentation to indicate the use of appropriate reporting guidelines for health-related research (see [EQUATOR Network](http://www.equator-network.org/%20)), life science research (see the [BioSharing Information Resource](https://biosharing.org/)), or the [ARRIVE guidelines](http://www.plosbiology.org/article/info:doi/10.1371/journal.pbio.1000412) for reporting work involving animal research. Where applicable, authors should refer to any relevant reporting standards documents in this form.

If you have any questions, please consult our Journal Policies and/or contact us: [editorial@elifesciences.org](mailto:editorial@elifesciences.org).

**Sample-size estimation**

- You should state whether an appropriate sample size was computed when the study was being designed
- You should state the statistical method of sample size computation and any required assumptions
- If no explicit power analysis was used, you should describe how you decided what sample (replicate) size (number) to use

Please outline where this information can be found within the submission (e.g., sections or figure legends), or explain why this information doesn’t apply to your submission:

For cryo-EM analysis, the number of micrographs collected and the number of picked particles are described in Materials and Methods and in Supplementary Figure 1 and Table S1.

**Replicates**

- You should report how often each experiment was performed
- You should include a definition of biological versus technical replication
- The data obtained should be provided and sufficient information should be provided to indicate the number of independent biological and/or technical replicates
- If you encountered any outliers, you should describe how these were handled
- Criteria for exclusion/inclusion of data should be clearly stated
- High-throughput sequence data should be uploaded before submission, with a private link for reviewers provided (these are available from both GEO and ArrayExpress)

Please outline where this information can be found within the submission (e.g., sections or figure legends), or explain why this information doesn’t apply to your submission:

In vitro binding and HMTase assays on recombinant nucleosomes:

Every nucleosome-binding and HMTase assay subjected to quantification as described in the figure legends and in Materials and Methods was performed in triplicate, in three independently performed experiments. The recombinant wild-type PRC2 and every mutant version of PRC2 used for these assays came from at least two independently expressed and purified batches of the complex.

ChIP-seq analysis:

For each genotype and developmental stage, the material (imaginal disc and CNS tissues from larvae, or embryos) was collected over a period of several days and larval tissues or embryos, respectively were then pooled to generate single large batches of chromatin that were then calibrated and used for multiple ChIP-seq experiments. With every antibody, ChIP experiments were performed in duplicates, and two independent libraries were generated and sequenced. The sequence datasets generated in this study have been deposited in GEO (accession number: GSE148254)

Link for reviewers: <https://www.ncbi.nlm.nih.gov/geo/query/acc.cgi?acc=GSE148254>
Enter token gvwxqqysdbcldgx into the box

Analysis of phenotypes in Drosophila H3K36R and H3K36A mutants:

For western blot analyses on larval or embryo extracts, large batches of extracts were generated by pooling larval tissues or hand-sorted embryos, respectively, that had been collected over multiple days. The material was then calibrated and western blots were performed in two technical replicates.

Every antibody staining experiment on embryos or larvae was repeated at least three times, in each case on independently collected batches of embryos and larvae of the appropriate genotype. The numbers of analyzed individuals of a given genotype and the numbers of individuals showing a particular phenotype described in the figures and figure legends represent the total numbers of analyzed individuals from different experiments.

**Statistical reporting**

- Statistical analysis methods should be described and justified
- Raw data should be presented in figures whenever informative to do so (typically when N per group is less than 10)
- For each experiment, you should identify the statistical tests used, exact values of N, definitions of center, methods of multiple test correction, and dispersion and precision measures (e.g., mean, median, SD, SEM, confidence intervals; and, for the major substantive results, a measure of effect size (e.g., Pearson's r, Cohen's d)
- Report exact p-values wherever possible alongside the summary statistics and 95% confidence intervals. These should be reported for all key questions and not only when the p-value is less than 0.05.

Please outline where this information can be found within the submission (e.g., sections or figure legends), or explain why this information doesn’t apply to your submission:

Cryo EM: In context of structure determination and validation all relevant statistical analyses were performed within the Relion and Phenix pipelines with no intervention from the authors.

Biochemistry: Statistics applied are described in the figure legends and in the Materials and Methods.

(For large datasets, or papers with a very large number of statistical tests, you may upload a single table file with tests, Ns, etc., with reference to sections in the manuscript.)

**Group allocation**

- Indicate how samples were allocated into experimental groups (in the case of clinical studies, please specify allocation to treatment method); if randomization was used, please also state if restricted randomization was applied
- Indicate if masking was used during group allocation, data collection and/or data analysis

Please outline where this information can be found within the submission (e.g., sections or figure legends), or explain why this information doesn’t apply to your submission:

No group allocation or randomization was used in this study.

**Additional data files (“source data”)**

- We encourage you to upload relevant additional data files, such as numerical data that are represented as a graph in a figure, or as a summary table
- Where provided, these should be in the most useful format, and they can be uploaded as “Source data” files linked to a main figure or table
- Include model definition files including the full list of parameters used
- Include code used for data analysis (e.g., R, MatLab)
- Avoid stating that data files are “available upon request”

Please indicate the figures or tables for which source data files have been provided:

In Table S2, source data is provided for the data shown in Figure 4 and Supplemental Figure S8.
